# A qualitative exploration of priorities for quality improvement amongst Aboriginal and Torres Strait Islander primary health care services

**DOI:** 10.1186/s12913-021-06383-7

**Published:** 2021-05-06

**Authors:** Karen Carlisle, Veronica Matthews (Quandamooka), Michelle Redman-MacLaren, Kristina Vine, Nalita Nungarrayi Turner (Anmatyerre/Jaru), Catrina Felton-Busch (Yangkaal/Gangalidda), Judy Taylor, Sandra Thompson, Donald Whaleboat (Meriam Le), Sarah Larkins, With the LEAP Learning Community

**Affiliations:** 1 College of Medicine and Dentistry, James Cook University, Townsville, Bebegu Yumba Campus, QLD Australia; 2 Australian Institute of Tropical Health and Medicine, James Cook University, Townsville, Bebegu Yumba Campus, QLD Australia; 3 University of Sydney, University Centre for Rural Health, Lismore, New South Wales Australia; 4 College of Medicine and Dentistry, James Cook University, Cairns, Nguma-bada Campus, QLD Australia; 5 Murtupuni Centre for Rural & Remote Health, James Cook University, Mt Isa, QLD Australia; 6 Western Australia Centre for Rural Health, University of Western Australia, Geraldton, Western Australia Australia

**Keywords:** Quality improvement, Primary health care, Aboriginal and Torres Strait Islander health

## Abstract

**Background:**

Achieving quality improvement in primary care is a challenge worldwide, with substantial gaps between best practice and actual practice. Within the context of Australia, Aboriginal and Torres Strait Primary Health Care (PHC) services have great variation across settings, structures and context. Research has highlighted how these contextual differences can critically influence the success of Quality Improvement (QI) interventions and outcomes. Less understood is the interaction between local context and other factors, which may impact the implementation of QI interventions. This paper aims to explore the strengths and challenges in QI for Aboriginal and Torres Strait Islander PHC services and their priorities for improvement.

**Methods:**

A multiple case study design was adopted, working with eight Aboriginal and Torres Strait Islander PHC services in Northern Territory, Queensland and Western Australia. Data were collected via a health service survey, semi-structured interviews with health service staff and service users and researcher observations, to explore QI and perceptions of care quality at the service level. Data reported here were analysed using an iterative thematic technique, within-case and across-case.

**Results:**

A total of 135 interviews were conducted with health service staff, service users and community members. Participants emphasised the centrality of resilient community, committed workforce and valued Aboriginal and Torres Strait Islander team members in delivering care. A shared purpose around improving the health of community was a significant driver. Key challenges included staff turnover and shortages, a complex and overwhelming acute and chronic care workload, building relationships and trust between health services and the community. Service-suggested priority areas for improvement were categorised into five themes: i) cultural safety (community driving health and planning for culturally safe services); ii) community engagement (through clinical activities in the community); iii) shared ownership and a team approach around QI; iv) strengthening systems and consistent ways of doing things in the health service; and v) strengthening local workforce (and resources for a culturally safe workforce).

**Conclusions:**

These findings advance understandings of relational, community and cultural factors which are identified priorities for the delivery of quality care in Aboriginal and Torres Strait Islander PHC services across varied contexts.

**Supplementary Information:**

The online version contains supplementary material available at 10.1186/s12913-021-06383-7.

## Background

Improving health outcomes for Aboriginal and Torres Strait Islander peoples is an important Australian health priority. Access to strong, accessible and appropriate comprehensive PHC is one strategy to achieve this goal, although much of the quality improvement (QI) literature to date refers to more limited primary care. Achieving improvement in the quality of primary care on a broad scale is a challenge worldwide, with evidence that there is a substantial gap between best practice as defined by clinical practice guidelines and actual practice [1]. Ten common “building blocks” of high-performing primary care are often cited, with three “ingredients” for transformation of data-driven improvements: incentives; technical assistance; and international and Australian tools to assess these [2, 3]. Yet, success in implementation of complex interventions to improve the quality of primary care is often patchy, with a 2016 systematic review finding that the “fit” between the intervention and the context was often critical in determining intervention success, but that few studies reported sufficiently on the interaction between context and other factors [4].

PHC services that serve Aboriginal and Torres Strait Islander communities are heterogeneous, with great variety in geographical setting, size, governance and organisational structure. The quality of care provided by such services, and the health outcomes achieved, also varies significantly [5, 6]. In response to QI, some services consistently achieve relatively high performance, due to an interplay between strong and stable organisations, good governance and clinical leadership [7]. Together with mechanisms to facilitate community engagement, these enable perseverance with participation in QI [6, 8]. In contrast, some services show limited improvement (or sometimes none), due to a range of interwoven implementation, resourcing and community contextual factors, often the inverse of those underlying high performance [9, 10]. To achieve Aboriginal and Torres Strait Islander population health impact, we need to understand how to strengthen the quality of care in PHC on a broad scale and broaden our discourse on quality as defined by the communities we serve [11].

Sustained use of QI in Aboriginal and Torres Strait Islander PHC can improve health care delivery [12]. Alongside responsiveness to community context and local needs [6, 8], research has pointed to enabling factors which can enhance implementation of QI in Aboriginal and Torres Strait Islander services. These include organisation-wide commitment to QI (including policy and resourcing) [13–15], QI leadership [15, 16], facilitation to support QI [17–19], strong community linkages [7], teamwork and a stable, well-prepared workforce [16, 20, 21].

**Fig. 1:**
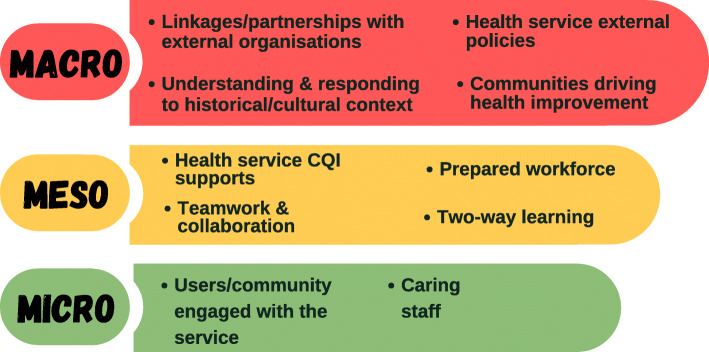
Factors influencing quality improvement (QI) at high-improving services [23]

Working with high-improving services to understand their secrets of success in achieving continuous high improvement in response to QI initiatives/activities, we found no statistically significant association between quality improvement status over time and service size, governance models or remoteness [22]. This suggests that multifarious workforce, organisational and resourcing factors alongside the wider community context combine to influence the degree to which service quality improves in response to QI cycles. Even amongst this group of high-improving services, the ways in which QI was conceived, implemented and communicated varied [23]. Qualitative exploration found themes such as: i) committed staff leadership (clinical and managerial); ii) strong partnerships within community and broader networks; and iii) embeddedness in Aboriginal and Torres Strait Islander culture, are common at the meso and micro systems level but not universal (Fig. 1). Each system has distinguishing interaction patterns (e.g. shared decision-making or staff relationships) that support quality improvement, and high-improving services are responsive to modify their activities according to context to optimise quality improvement. For example, in jurisdictions with unsupportive macro policies it appears that impetus is gained through generating local solutions to overcoming challenges, and ensuring that they are implemented with due attention to culture [11].

Following this work, we are now exploring barriers to quality improvement and strategies to address these in the Leveraging Effective Ambulatory Practices (LEAP) project. This paper explores the key implementation challenges for quality improvement at eight Aboriginal and Torres Strait Islander PHC services and how they interact, along with service-suggested strategies for improvement.

## Methods

### Approach

The study employed a comparative case study design, collecting qualitative data from eight sites across northern Australia to identify key implementation challenges in reaching quality goals [24]. The overarching framework for this study drew on Indigenous research methodologies, guided by “Indigenous worldviews, perspectives, values and lived experience as their central axis” [25]. This approach was operationalised through attention to appropriate methods (yarning), reciprocity and relationships between community members and interviewers, striving to ensure respect for Aboriginal and Torres Strait Islander cultures, foregrounding community voices, and acknowledging the social, historical and political context [26]. A strengths-based participatory approach informed the design of the study and included the creation of a Learning Community (LC), involving PHC staff from participating services and investigators. Aboriginal and Torres Strait Islander researchers and participants were involved at all stages.

### Setting and sampling strategy

Invitations to self-nominate for the study were distributed to Aboriginal and Torres Strait Islander PHC services in Queensland (QLD), Northern Territory (NT) and Western Australia (WA) via existing networks. Inclusion criteria were: i) community controlled or Government PHC service; ii) involved in QI audits for three or more years; iii) not meeting own QI goals; and iv) consent from management/board and staff.

Eight self-selected PHC services represented a range of jurisdictions, service sizes, remoteness, governance models and approaches to QI. In Australian Aboriginal and Torres Strait Islander PHC services, a number of CQI tools and processes are in use. These include the Northern Territory (NT) Chronic Condition Management Model (CCMM), NT Aboriginal Health Key Performance Indicators (NT AHKPIs), the Queensland Aboriginal and Islander Health Council (QAIHC) Core Indicators reports (from PENCAT tools), ABCD/One21seventy audit tools and the National Indigenous KPIs [27–29]. The characteristics of participant self-nominated services and their communities are described in Table 1.

**Table 1:** Characteristics of participant Aboriginal and Torres Strait Islander PHC services and their service populations

Health Service	Governance	State	Rurality (AGSC)	Population	% of Aboriginal and/or Torres Strait Islander population ^a^	QI Tools
**PHC service 1**	Government operated	QLD	Very Remote	1000–10,000	82.4	One21seventy and NKPIs
**PHC service 2**	ACCHS	QLD	Regional	> 100,000 (Service population approx.. 9000)	7.9	QAIHC PENCAT Tools and NKPIs
**PHC service 3**	ACCHS	QLD	Very Remote	1000–10,000	12.7	QAIHC PENCAT Tools and NKPIs
**PHC service 4**	ACCHS	NT	Very Remote	< 1000	94	NT AHKPIs One21seventy
**PHC service 5**	Government operated	NT	Very Remote	< 1000	88.6	NT AHKPIs, NT CCMM and One21seventy
**PHC service 6**	ACCHS	NT	Very Remote	< 1000	89.3	NT AHKPIs, NT CCMM and One21seventy
**PHC service 7**	Government operated	NT	Remote	< 1000	81.6	NT AHKPIs, NT CCMM and One21seventy
**PHC service 8**	ACCHS	WA	Very Remote	1000–10,000	Approx. 90%	One21seventy, NKPIs

^a^ABS 2016 census data

Most of the services are situated in remote locations, with two services based in larger regional centres. Five services are Aboriginal Community Controlled Health Services (ACCHS); governed by the local Aboriginal community through an elected Board of Management to deliver holistic and culturally appropriate health care to the community [30]. Three services are operated by the relevant state health department.

### Data collection

Case studies in each of the PHC services involved two members of the research team, (including at least one Aboriginal or Torres Strait Islander researcher) visiting the service from late 2018 to early 2019. To enhance validity of findings three data sets were collected with findings triangulated [24].

- PHC service survey, noting governance, service delivery and staffing profile;
- Semi-structured interviews and/or focus groups with health service staff and health service users;
- Observation of service operations and staff participation in the QI process, captured as reflections from visiting researchers.

Data collection tools (PHC service survey and semi-structured interview guides) were co-designed by the LEAP LC at a face-to-face meeting in 2018, prior to data collection.

See Supplementary File 1 for PHC service information survey and semi-structured interview guides.

Health service staff and service users were informed of the research, including dates of the research team visit to the health service and invited to take part in an interview or focus group. This information was shared via the local health service champion/main contact for the research team. It was stressed that taking part in the research was voluntary and individuals could decline to participate without penalty.

Service information surveys were completed via structured interviews with the PHC service manager. Semi-structured interviews with local and visiting staff and managers explored how QI is working at their PHC service including enablers and barriers, the workforce and their role in QI, community involvement in QI and ideas for improving quality of care. Aboriginal and Torres Strait Islander community members were asked about their use of the service, their perspectives about care quality, and whether they had opportunities to provide feedback to improve care. A small number of focus groups (*N* = 6) were conducted at the request of staff and service users. Two focus groups were conducted with health service users and four focus groups with health service staff; groups were comprised of either all health service staff or all health service users, with no mixed groups. The number of participants in the focus groups varied: 10 service users attended one focus group; and the remaining groups ranged from two to four participants. The semi-structured interview guide was used to facilitate the focus groups. Duration of the focus groups ranged from 20 to 40 min. All interview and focus group participants provided informed written consent.

#### Ethics

Human research ethics approval was obtained from Northern Territories Department of Health and Menzies School of Health Research (2018–3064), Western Australian Aboriginal Health Ethics Committee (884), Queensland Health (HREC/QCH/43490) and James Cook University (H7390).

### Data analysis

Digital recordings were transcribed verbatim and interview notes and observations from the visiting researchers were imported into NVivo12 (QSR International) software. Data were analysed using an abductive approach which is an inferential process to identify and interpret patterns of meanings and explore novel concepts emerging from the data [31]. Within case analysis was conducted first to explore implementation challenges to improvements in quality of care at each service and factors about the health system and context that interacted with implementation of QI. Initial codes were identified, reviewed and connected into themes with patterns of meaning checked across data in each case study. The within case themes were the basis of draft individual service summary reports, called “Improvement stories”.

Following completion of the draft stories, each PHC service was revisited by the researchers to ensure that interpretations reflected the deep contextual knowledge and experience of the interviewees. Changes were made to the stories as a result of discussions, consistent with participatory approaches and PHC services as co-inquirers.

Across case analyses comprised of reviewing themes from each study site and identifying common themes related to strengths, challenges and priority areas for quality improvement. Sub-themes were identified directly from each of the service summary reports, and then grouped into overarching themes. These priority areas of improvement were presented to the LEAP LC at a second face-to-face meeting in 2019. Here, participants were invited to take part in a series of Yarning Circles which reflected on priority areas of improvement, their experiences and ideas which were subsequently incorporated into the improvement stories.

## Results

A total of 135 participants took part in interviews and focus groups across the eight case study PHC services (Table 2). Almost one quarter were health service users/members of the community (*n* = 31). Health service staff interviewed included Aboriginal Health Practitioners/Workers (AHPs/AHWs) (*n* = 23), other clinical staff (*n* = 61), and non-clinical staff and managers (*n* = 20). Approximately two-thirds of interviewees were Aboriginal and/or Torres Strait Islander (*n* = 84).

**Table 2:** Characteristics of interviewees from eight participating PHC services

Health Service	Health Service Staff	Community/ Health Service User^a^	Aboriginal and/or Torres Strait Islander	Non-Indigenous	Female	Male	Total
**PHC service 1**	11	3	10	4	12	2	14
**PHC service 2**	18	2	14	6	17	3	20
**PHC service 3**	16	17	31	3	23	11	34
**PHC service 4**	12	2	10	4	9	5	14
**PHC service 5**	9	0	4	5	4	5	9
**PHC service 6**	13	0	4	9	9	4	13
**PHC service 7**	7	2	6	3	6	3	9
**PHC service 8**	18	5	5	17	15	7	22
**Total**	104 (77%)	31 (23%)	84 (62%)	51 (38%)	95 (71%)	40 (29%)	135

^a^Including board members

Challenges and strengths of service approaches to QI activities are summarised in Table 3. Participants emphasised the strengths of resilient community, and the quality and commitment of their health workforce and teamwork in delivering care. Aboriginal and Torres Strait Islander team members and their contributions were highly valued. Support for QI from jurisdictional peak bodies and shared purpose around improving the health of community was important. Challenges can be summarised in terms of staff turnover and shortages, complex and overwhelming acute and chronic care workloads, challenges in building relationships and trust between the clinic and the community and a lack of local ownership of QI efforts. Additional issues affecting systematic application of QI included adequate IT systems and housing to support stability of local remote workforce.

**Table 3:** Challenges and strengths of service approaches to QI activities identified in PHC services

	Challenges to QI	Strengths of service approaches to QI
**PHC service 1**	Coordination of multiple specialist visits (unrealistic expectations) IT system transitions Uncertainty about future service provision Staff shortage (IHWs) (especially male) Unclear ownership of QI Lack of space/rooms	Strong sense of team amongst workers Ethos of quality care and “keeping the door open” Recognition of importance of “working culture way” Links with community (through staff)
**PHC service 2**	Issues with patient flow – no-shows, waiting time and transport Rapid growth and diversification Communication challenges between external services/between Board and staff	Strong systems and active QI implementation Long term staff, strong teams and links with community Clients comfortable and perceive quality care Quality, holistic care provision
**PHC service 3**	Understanding of QI processes (QI as “scary words”) QI happening but not evidenced or measured Engaging patients to come in Managing referrals and visiting teams Busyness!	Ethos and values of quality care AHW led service Strong leadership and committed workforce Community outreach Effective use of IT systems Open communication
**PHC service 4**	Working across languages Lack of male AHPs Burden on AHPs - tension between cultural expectations & health service delivery Some people not attending clinic Limited understanding of audit and QI Ageing workforce	Culture-embedded care delivered by local Aboriginal staff Majority Aboriginal staff – valued AHP workforce Collaborative workforce Good community engagement
**PHC service 5**	Staff turnover/shortages and challenges recruiting and training AHPs Limited local cultural orientation for new staff Large complex clinical workload impacting on turnover and continuity of care Building trust with community/disconnect between clinic and community Remoteness and isolation Complex health service delivery arrangements	AHPs play an important role in care delivery Committed staff with relationships with community Strong links with AMSANT and support IT systems used to support care Support for staff training and upskilling
**PHC service 6**	Acute care demands “like constantly chasing your tail” Importance of peak body support Staff turnover and the “departure lounge” Lack of AHP and cross-cultural communication Links between community members and clinic staff	Resilient community Quality staff
**PHC service 7**	Perceived “top down” approach to QI and staff not feeling they had a say in QI Local staff feel they are always on call Not fully utilizing knowledge of local staff	Strong stable Aboriginal workforce, valued within service Receptive community Health systems support QI by all staff
**PHC service 8**	QI not yet embedded in the organizational culture Large clinical load Geographic isolation (incl. connectivity) Staff turnover; lack of AHP (especially males) Limited external support for QI	Strong shared motivation to improve health Support for QI from management

Suggested priority areas for improvement raised by participants were categorised into five themes: i) cultural safety (shared ownership and planning for culturally safe services); ii) community engagement (through clinical activities in the community); iii) shared ownership and a team approach around quality improvement; iv) strengthen systems and consistent ways of doing things in the health service; and v) strengthening local workforce (and resources). Table 4 summarises subthemes and ideas raised by participants.

**Table 4:** Suggested priority areas and strategies for improvement

1. **Cultural safety– shared ownership and planning for culturally safe services (8 services)** “*we use culture, and balance with Balanda ways to health*” •Strengthen cultural safety of the organisation – training for new staff (by local Elders) and learn language •More local Aboriginal and Torres Strait Islander staff in all kinds of roles within the service •Better understanding of local Aboriginal or Torres Strait Islander culture and building relationships •Listen to communities to gather ideas for change •Share ownership of CQI process with community (community driven health care) •Use more visual means to communicate with community e.g. photos to strengthen two-way communication	
2. **Community engagement (8 services) – take clinical activities out to community** “*sitting down with the community*” •Increase outreach services and staff outreach roles – getting health out of the clinic into community • “Healthy clinic”/health camps in community for two-way learning •Strengthen linkages with other clinical/linked services in communities •Get involved in community events and through existing groups such as women’s groups •Engage those who don’t attend clinics, through home visits	
3. **Shared ownership and team approach around quality improvement (8 services)** ”*Probably just give us more - a bit more autonomy again. I remember the early years, we had that autonomy and you could do things that you saw in your particular community that might be of benefit rather than a general this is going to happen everywhere sort of thing.”* •All staff involved and supported to have a say with local autonomy •Quality improvement is normal and systematised •Regular communication and meetings to workshop issues around workload and QI •Education and training around CQI to increase understanding of all staff •Mentor staff new to CQI processes •Leadership to support teamwork and engagement around shared vision	
4. **Strengthen systems and ways of doing things in the health service (8 services)** *Consistent “ways of doing things …*.” ***“****It requires a person to be able to take charge of it and action the recalls and [manage the] system. It’s so much more than just the software.. It’s about having … .staff to own it and run it.”* •Build consistency and systems in face of changing staff •Improving patient flow and transport •Take control of referrals and external clinics e.g. visiting specialists •Strengthen use of IT systems •Joined up planning of services to meet needs	
5. **Strengthening local workforce (and resources) (5 services but strong intersection with cultural security and community engagement)** *“So from an organisation, we need to look at that, how we can change that ethos and have more health workers”*. *“Aboriginal Health Workers would* “*hold your hand and guide you through. They can be the element of change.”* •More Aboriginal and Torres Strait Islander staff at all levels of service •Increase training and support for IHWs (esp. male) •Sustainable and flexible workforce models •Succession planning for workforce •More vehicles and office space, with health centres designed to be welcoming, new clinic	

## Discussion

This study involving eight PHC services in regional and remote settings adds to our understanding of how common system factors interact and affect responses to QI within PHC across Australia. Self-identified improvement priorities focused on strengthening community engagement and cultural security [11] in addition to the more regularly reported on factors such as teamwork or shared mission and systems strengthening [16, 20–23]. The relational, trust and cultural aspects of care were vital to quality as perceived by these individuals and communities [11, 32]. Unsurprisingly, given the context, workforce was raised as a common priority; however the focus was broad and intersected strongly with the cultural safety of the organisation, trust in care provided and continuity of care, rather than the financial and systems imperatives inherent in turnover in other studies [33]. Two main elements related to: 1) increasing the numbers and prominence of the Aboriginal and Torres Strait Islander workforce in a range of clinical and non-clinical roles within the service and; 2) strengthening the stability of the health workforce and cultural safety of their workplaces. This is similar to factors that have been highlighted in the participatory development of an Aboriginal model of care (an Aboriginal health care home) in East Arnhem Land [34].

Strengths of the PHC services identified by staff and community provide evidence of the immutable interconnection between culture, community and the delivery of quality care and builds on what we know about two way learning and community driving health [11, 23]. Critical to a “strong” PHC service response to quality improvement was an integrated Aboriginal and Torres Strait Islander workforce that (teamwork) that were supported (through appropriate training, QI systems and leadership) and valued in their provision of quality care to the community [10, 35]. These factors, including the esteem in which Aboriginal staff are held and the valuing of their input have also been identified as critical in high performing tertiary health services [36]. Other important factors identified by service staff are particularly relevant for health services working with community. The importance of cultural awareness training for staff [37, 38], being prepared to slow down in the process of community engagement and providing choice for service users in terms of who and how they engage with the health service [39]. Finally, recognising that there needs to be sharing both ways, exchanging information and feedback between the health service and community [11, 23].

Critically, this study has shown that the involvement of PHC service staff and community members shifted thinking away from a deficit model to solutions focused on service and community strengths, for example strengthening cultural safety and links in community. Furthermore, findings add to the strong body of literature suggesting that the motivation to improve clinical practice can be strengthened through facilitated, decision making involving Aboriginal and Torres Strait Islander staff and community to strengthen understanding of pathways to change, [7, 37] but that these need broadening to co-led community design of improvement strategies for maximal impact [34].

The methodology adopted by the research team can help understand ways to work in partnership- with local health workforce, community members and service users in the development of appropriate data collection tools, collection and interpretation of the data. It reinforces the message that improved quality of care in this context will only occur if end users are intimately involved in discussions about what is important [40]. Our research approach based on two-way learning with Aboriginal and Torres Strait Islander and non-Indigenous researchers working together with communities and partners at all stages was vital in achieving integrity in collection and interpretation of data. Combining the interpretation contributes to rigor, brings multiple perspectives, and facilitates a richer understanding of quality in PHC and priorities of the Aboriginal and Torres Strait Islander communities with whom we work [11, 40]. Perspectives from the wider membership of the LC contributed to this interpretative richness [23].

### Study strengths and limitations

Strengths of this study included an appropriate choice of methodologies to maximise two-way learning through inclusion of both an Aboriginal or Torres Strait Islander and a non-Indigenous researcher for all community visits. An experienced team, case study design, liberal use of data and coder triangulation and the prioritisation of Indigenous voices through the LC at all stages of design and analysis contributed to trustworthiness of the findings. Limitations include the short amount of time researchers could spend in each community that impacted relationship building outside of health services and community input to service summary reports. In addition, only eight Indigenous PHC services were involved, although their distribution over regional and remote settings in three jurisdictions gives some confidence that findings are likely to be transferable to a broader range of settings.

## Conclusions

To continue to strengthen the quality of primary health care available to Aboriginal and Torres Strait Islander peoples in Australia, it is vital that we broaden our understanding of quality to include the relational, cultural and trust components highlighted as priorities for improvement. Respectful attention in partnership to address these “non-clinical” indicators of quality care, in addition to action on the broader social, environmental and cultural determinants of health are all vital to “Close the Gap”.

## Supplementary Information

**Additional file 1.**

## Data Availability

The de-identified datasets used and analysed during the current study are available from the corresponding author on reasonable request.

## Abbreviations

ACCHS: Aboriginal Community Controlled Health Services
AHP: Aboriginal Health Practitioner
AHW: Aboriginal Health Worker
LC: Learning Community
LEAP: Leveraging Effective Ambulatory Practices
NT: Northern Territory
PHC: Primary Health Care
QI: Quality Improvement
QLD: Queensland
WA: Western Australia

## References

[1] Mickan S, Burls A, Glasziou P (2011). Patterns of “leakage” in the utilisation of clinical guidelines: a systematic review. Postgrad Med J.

[2] Bodenheimer T, Ghorob A, Willard-Grace R, Grumbach K (2014). The 10 building blocks of high-performing primary care. Ann Fam Med.

[3] Crossland L, Janamian T, Sheehan M, Siskind V, Hepworth J, Jackson CL (2014). Development and pilot study of the primary care practice improvement tool (PC-PIT): an innovative approach. Med J Aust.

[4] Lau R, Stevenson F, Ong BN, Dziedzic K, Treweek S, Eldridge S, et al. Achieving change in primary care—causes of the evidence to practice gap: systematic reviews of reviews. Implement Sci. 2015;11(1):40. 10.1186/s13012-016-0396-4 PMC4802575 27001107

[5] Si D, Bailie R, Dowden M, Kennedy C, Cox R, O'Donoghue L, Liddle H, Kwedza R, Connors C, Thompson S, Burke H, Brown A, Weeramanthri T (2010). Assessing quality of diabetes care and its variation in Aboriginal community health centres in Australia. Diabetes Metab Res Rev.

[6] Matthews V, Schierhout G, McBroom J, Connors C, Kennedy C, Kwedza R, Larkins S, Moore E, Thompson S, Scrimgeour D, Bailie R (2014). Duration of participation in continuous quality improvement: a key factor explaining improved delivery of type 2 diabetes services. BMC Health Serv Res.

[7] Schierhout G, Hains J, Si D, Kennedy C, Cox R, Kwedza R, O’Donoghue L, Fittock M, Brands J, Lonergan K, Dowden M, Bailie R (2013). Evaluating the effectiveness of a multifaceted, multilevel continuous quality improvement program in primary health care: developing a realist theory of change. Implement Sci.

[8] Peiris D, Brown A, Howard M, Rickards B, Tonkin A, Ring I (2012). Building better systems of care for Aboriginal and Torres Strait islander people: findings from the Kanyini health systems assessment. BMC Health Serv Res.

[9] Bond C, Brough M, Willis J, Stajic J, Mukandi B, Canuto C, Springer S, Askew D, Angus L, Lewis T (2019). Beyond the pipeline: a critique of the discourse surrounding the development of an indigenous primary healthcare workforce in Australia. Australian J Primary Health.

[10] Jongen C, McCalman J, Campbell S, Fagan R (2019). Working well: strategies to strengthen the workforce of the indigenous primary healthcare sector. BMC Health Serv Res.

[11] Turner N, Taylor J, Larkins S, Carlisle K, Carter M, Thompson S, Bailie R (2019). Conceptualising the association between community participation and continuous quality improvement in Aboriginal and Torres Strait islander primary health care services. Qual Health Res.

[12] Laycock A, Conte K, Harkin K, Bailie J, Matthews V, Cunningham F, Ramanathan SA, Bailie R (2019). Improving the quality of primary health care for Aboriginal and Torres Strait Islander Australians.

[13] Bailie R, Matthews V, Larkins S, Thompson S, Burgess P, Weeramanthri T, Bailie J, Cunningham F, Kwedza R, Clark L (2017). Impact of policy support on uptake of evidence-based continuous quality improvement activities and the quality of care for Indigenous Australians: a comparative case study. BMJ Open.

[14] Gardner K, Dowden M, Togni S, Bailie R (2010). Understanding uptake of continuous quality improvement in indigenous primary health care: lessons from a multi-site case study of the audit and best practice for chronic disease project. Implement Sci.

[15] Schierhout G, Brands J, Bailie R (2010). Audit and Best Practice for Chronic Disease Extension Project 2005–2009: Final Report.

[16] Newham J, Schierhout G, Bailie R, Ward PR (2016). ‘There’s only one enabler; come up, help us’: staff perspectives of barriers and enablers to continuous quality improvement in Aboriginal primary health-care settings in South Australia. Australian J Primary Health.

[17] Laycock A, Harvey G, Percival N, Cunningham F, Bailie J, Matthews V, Copley K, Patel L, Bailie R (2018). Application of the i-PARIHS framework for enhancing understanding of interactive dissemination to achieve wide-scale improvement in indigenous primary healthcare. Health Res Policy Syst.

[18] Cunningham FC, Ferguson-Hill S, Matthews V, Bailie R (2016). Leveraging quality improvement through use of the systems assessment tool in indigenous primary health care services: a mixed methods study. BMC Health Serv Res.

[19] Stoneman A, Atkinson D, Davey M, Marley J (2014). Quality improvement in practice: improving diabetes care and patient outcomes in Aboriginal community controlled health services. BMC Health Serv Res.

[20] Marley J, Nelson C, O’Donnell V, Atkinson D (2012). Quality indicators of diabetes care: an example of remote-area Aboriginal primary health care over 10 years. Med J Aust.

[21] Bailie J, Laycock A, Matthews V, Bailie R (2016). System-level action required for wide-scale improvement in quality of primary health care: synthesis of feedback from an interactive process to promote dissemination and use of aggregated quality of care data. Front Public Health.

[22] Larkins S, Woods C, Matthews V, Thompson S, Schierhout G, Mitropoulos M, Patrao T, Panzera A, Bailie R (2016). Responses of Aboriginal and Torres Strait islander primary health care services to continuous quality improvement (CQI) initiatives. Front Public Health.

[23] Larkins S, Carlisle K, Taylor J, Turner N (2019). “At the grass roots level it’s about sitting down and talking”: Exploring quality improvement through case studies with high-improving Aboriginal and Torres Strait Islander primary health care services. BMJ Open.

[24] Yin RK (1994). Case study research: design and methods.

[25] Walter M, Suina M (2019). Indigenous data, indigenous methodologies and indigenous data sovereignty. Int J Soc Res Methodol.

[26] Martin K (2003). Ways of knowing, being and doing: a theoretical framework and methods for indigenous and Indigenist research. J Australian Stud.

[27] National Aboriginal Community Controlled Health Organisations (2013). Healthy for life: Aboriginal community controlled health services. Report card. IHW 97.

[28] Queensland Aboriginal and Islander Health Council (2014). External Report 3 – Aboriginal and Islander Community Controlled Health Services Clinical Excellence (ACE) Program.

[29] Burgess CP, Sinclair G, Ramjan M, Coffey PJ, Connors CM, Katekar LV (2015). Strengthening cardiovascular disease prevention in remote indigenous communities in Australia's Northern Territory. Heart, Lung Circulation.

[30] Panaretto K, Wenitong M, Button S (2014). Aboriginal community controlled health services: leading the way in primary care. Med J Aust.

[31] Timmermans S, Tavory I (2012). Theory construction in qualitative research: from grounded theory to abductive analysis. Sociological Theory.

[32] Davy C, Cass A, Brady J, DeVries J, Fewquandie B, Ingram S, Mentha R, Simon P, Rickards B, Togni S, Liu H. Facilitating engagement through strong relationships between primary healthcare and Aboriginal and Torres Strait Islander peoples. Aust N Z J Public Health. 2016;40(6):535–41. 10.1111/1753-6405.12553 27523395

[33] Zhao Y, Russell D, Guthridge S, Ramjan M, Jones M, Humphreys J, Wakerman J (2019). Costs and effects of higher turnover of nurses and Aboriginal health practitioners and higher use of short-term nurses in remote Australian primary care services: an observational cohort study. BMJ Open.

[34] Smith G, Kirkham R, Gunabarra C, Bokmakarray V, Burgess CP (2018). “We can work together, talk together”: an Aboriginal health care home. Aust Health Rev.

[35] Wakerman J, Humphreys JS (2013). Sustainable workforce and sustainable health systems for rural and remote Australia. Med J Aust.

[36] Taylor EV, Lyford M, Parsons L, Mason T, Sabesan S, Thompson SC (2020). “We’re very much part of the team here”: a culture of respect for Indigenous health workforce transforms Indigenous health care. PLoS ONE.

[37] Laverty M, McDermott DR, Calma T (2017). Embedding cultural safety in Australia’s main health care standards. Med J Aust.

[38] Gubhaju L, Williams R, Jones J, Hamer D, Shepherd C, McAullay D, Eades SJ, McNamara B (2020). “Cultural security is an On-Going journey …” Exploring views from staff members on the quality and cultural security of services for Aboriginal families in Western Australia. Int J Environ Res Public Health.

[39] Woods C, Larkins S, Carlisle K, et al. Exploring systems that support good clinical care in indigenous primary health care services: a retrospective analysis of longitudinal systems assessment tool data from high improving services. Front Public Health. 2017;5. 10.3389/fpubh.2017.00045. 10.3389/fpubh.2017.00045 PMC5364947 28393064

[40] Harfield S, Pearson O, Morey K, Kite E, Canuto K, Glover K, Gomersall JS, Carter D, Davy C, Aromataris E, Braunack-Mayer A (2020). Assessing the quality of health research from an indigenous perspective: the Aboriginal and Torres Strait islander quality appraisal tool. BMC Med Res Methodol.

