# Supplementary material for: A qualitative exploration of priorities for quality improvement amongst Aboriginal and Torres Strait Islander primary health care services
Source: BMC Health Serv Res. 2021 May 6;21:431. doi: 10.1186/s12913-021-06383-7 (PMC8101223; doi:10.1186/s12913-021-06383-7)
Supplement: Supplementary file 1 — Additional file 1. [file 12913_2021_6383_MOESM1_ESM.docx]

**A qualitative exploration of priorities for quality improvement amongst Aboriginal and Torres Strait Islander primary health care services**

Corresponding author: Karen Carlisle, College of Medicine and Dentistry, James Cook University, Townsville, QLD, Australia. Australian Institute of Tropical Health and Medicine, James Cook University, Townsville, QLD, Australia. [karen.carlisle@jcu.edu.au](mailto:karen.carlisle@jcu.edu.au)

Veronica Matthews, University Centre for Rural Health, University of Sydney, Lismore, New South Wales, Australia. veronica.matthews@sydney.edu.au

Michelle Redman-MacLaren, College of Medicine and Dentistry, James Cook University, Townsville, QLD, Australia. Australian Institute of Tropical Health and Medicine, James Cook University, Townsville, QLD, Australia. michelle.maclaren@jcu.edu.au

Kris Vine, University College of Medicine and Dentistry, James Cook University, Townsville, QLD, Australia. kris.vine@jcu.edu.au

Nalita Turner, College of Medicine and Dentistry, James Cook University, Townsville, QLD, Australia. nalita.turner@jcu.edu.au

Catrina Felton-Busch, Centre for Rural & Remote Health, James Cook University, Mt Isa, QLD, Australia. Australian Institute of Tropical Health and Medicine, James Cook University, Townsville, QLD, Australia. catrina.feltonbusch@jcu.edu.au

Judy Taylor, College of Medicine and Dentistry, James Cook University, Townsville, QLD, Australia. Australian Institute of Tropical Health and Medicine, James Cook University, Townsville, QLD, Australia. judy.taylor@jcu.edu.au

Sandra Thompson, Western Australia Centre for Rural Health, University of Western Australia, Geraldton, Western Australia, Australia. [sandra.thompson@uwa.edu.au](mailto:sandra.thompson@uwa.edu.au)

Donald Whaleboat, College of Medicine and Dentistry, James Cook University, Townsville, QLD, Australia. donald.whaleboat@jcu.edu.au

Sarah Larkins, College of Medicine and Dentistry, James Cook University, Townsville, QLD, Australia. Australian Institute of Tropical Health and Medicine, James Cook University, Townsville, QLD, Australia. sarah.larkins@jcu.edu.au

With the LEAP Learning Community

**Supplementary File 1**

**PHC service information survey**

|  | **Section One: Health Centre Information** | | | | | | |
| --- | --- | --- | --- | --- | --- | --- | --- |
| **1.1** | Health Centre ID | _ _ _ _ _ _ _ _ | | **1.2** Survey Date | | / / | |
| **1.3** | Author’s initial and surname | | | | |  | |
| **1.3a** | **Informant’s name and role** | | | | |  | |
| **1.4** | **Governance. The Health Centre is** *(circle one option)***:** | | | | |  | |
|  | A NACCHO affiliated Aboriginal community controlled health service (ACCHS) | | | | | 1 | |
|  | A state/territory government service | | | | | 2 | |
|  | Other *(please specify)* | | | | | 3 | |
| **1.5** | How many **Outstations** or **Outlying Clinics** are serviced by the Health Service? | | | | |  | |
| **1.6** | **Accreditation. Is the Health Service accredited?** *(circle one option)***:** | | | | |  | |
| **1.6a** | Clinical accreditation Yes No | | | | | 1– AGPAL  2- RACGP | |
| **1.6b** | Organisational accreditation Yes No | | | | | 1. ISO 2. QIC | |
| **1.7** | **Are Electronic Medical Record systems used in this practice?** | | | | | Yes _1_ No _2_ | |
| **1.7a** | **If yes, which Electronic Medical Record systems are used in this health centre?**  *(circle all that apply)***:**  **Ferret**  **Best Practice**  **Communicare**  **Medical Director**  **MMDX**  **Other** *(please specify)* | | | | | 1  2  3  4  5  6 | |
| **1.8** | **If Electronic Medical Records are used, are they used for:** | | | | |  | |
| **1.8a** | Patient records | | | | | Yes _1_ No _2_ | |
| **1.8b** | Prescribing | | | | | Yes _1_ No _2_ | |
| **1.8c** | Recalls and reminders | | | | | Yes _1_ No _2_ | |
| **1.8d** | Other *(please specify)* | | | | | Yes _1_ No _2_ | |
| **1.9** | **Is this a teaching site?** | | | | | Yes _1_ No _2_ | |
|  | **If yes, the site is involved with teaching:** | | | | |  | |
| **1.9a** | Aboriginal and Torres Strait Islander Health Workers and/OR Registered Aboriginal Health Practitioners? (*please tick response)* | | | | | Yes _1_ No _2_ | |
| **1.9b** | If yes, please describe frequency  *(please specify)* | | | | | Weekly _1_  F/nightly _2_  Monthly _3_  Other _4_ _4_ | |
| **1.9c** | If yes, please describe duration  *(please specify)* | | | | | ≤1hr _1_  1-3 hrs _2_  All day _3_  Other _4_ | |
| **1.9d** | Nursing students? (*please tick response)* | | | | | Yes _1_ No _2_ | |
|  | If yes, please describe the frequency  *(please specify)* | | | | | Weekly _1_  F/nightly _2_  Monthly _3_  Other _4_ _4_ | |
| **1.9e** | If yes, please describe the duration  *(please specify)* | | | | | ≤1hr _1_  1-3 hrs _2_  All day _3_  Other _4_ | |
| **1.9f** | Medical students? (*please tick response) What year level?* | | | | | Yes _1_ No _2_ | |
| **1.9g** | If yes, please describe frequency  *(please specify)* | | | | | | Weekly _1_  F/nightly _2_  Monthly _3_  Other _4_ _4_ |
| **1.9h** | If yes, please describe the duration  *(please specify)* | | | | | ≤1hr _1_  1-3 hrs _2_  All day _3_  Other _4_ | |
| **1.9i** | Other students *(please specify)* | | | | | Yes _1_ No _2_ | |
| **1.9j** | If yes, please describe the frequency  *(please specify)* | | | | | Weekly _1_  F/nightly _2_  Monthly _3_  Other _4_ _4_ | |
| **1.9k** | If yes, please describe the duration  *(please specify)* | | | | | ≤1hr _1_  1-3 hrs _2_  All day _3_  Other _4_ | |
| **1.10g** | **How often do staff participate in professional development activities or does formal in-service training occur at this site?**    *(please specify)* | | | | | Weekly _1_  F/nightly _2_  Monthly _3_  Other _4_ _4_ | |
| **1.10h** | **Is professional development/staff training conducted internally or externally? (i.e. by service staff or by outside staff)** | | | | | Internal _1_  External _2_  Both _3_ | |
| **1.10i** | **How is professional development/staff training supported at this site?** | | | | | | |
| **1.11** | **CQI History – The Health Centre has participated in the following CQI audits:** | | | | |  | |
| 1.11a | **What Quality Improvement or Clinical Audit activities tools are used at this service? (e.g PENCAT tools, Improvement Foundation, Primary Care Collaboratives etc.)** | | | | | | |
| **1.12a** | Diabetes – if yes, from 20__ __ to 20__ __ | | | | | Yes _1_ No _2_ | |
| **1.12b** | Maternal Health – if yes, from 20__ __ to 20__ __ | | | | | Yes _1_ No _2_ | |
| **1.12c** | Child Health – if yes, from 20__ __ to 20__ __ | | | | | Yes _1_ No _2_ | |
| **1.12d** | Preventative – if yes, from 20__ __ to 20__ __ | | | | | Yes _1_ No _2_ | |
| **1.12e** | Vascular and Metabolic Syndrome – if yes, from 20__ __ to 20__ __ | | | | | Yes _1_ No _2_ | |
| **1.12f** | Who conducts the CQI audits?  How much continuity has there been over the period of time doing the ABCD audits? | | | | | | |
| **1.12g** | Who manages/oversees the CQI audits? | | | | | | |
| **1.12h** | How does your service analyse CQI data for system improvement?  Who participates in this process? | | | | | | |
| **1.12i** | How are goals for improvement set and shared? | | | | | | |
| **1.12j** |  | | | | | Yes _1_ No _2_ | |
| **1.13** | **Are any client satisfaction data collected? (yes/no) If yes, please specify:** | | | | | | |
|  | **Section Two: Reference Population – ABS 2011 Census statistics** | | | | |  | |
| **2.1** | **ASGS-RA Classification:** | RA1 _1_ RA2 _2_ RA3 _3_ RA4 _4_ RA5 _5_ | | | | | |
| **2.1a** | **Location. Where is the Health Service located** *(circle one option)***:** | | | | | | |
|  | Regional town (a settlement of <25,000 people where more than half of the residents are non-Indigenous, and which has a range of basic services in addition to a health centre) | | | | | | 1 |
|  | Remote Indigenous community (a settlement primarily inhabited by Indigenous people, which is in a regional/remote location; which is not in close proximity (less than 10km) to a regional town) | | | | | | 2 |
|  | Other *(please specify)* | | | | | | 3 |
| **2.1b** | What regular transport and accessibility options are available for residents?  *(please specify)* | | | | | Road _1_  Light Plane _2_  Barge/Boat _3_  Other _4_ | |
| **2.1c** | What emergency transport and accessibility options are available?  *(please specify)* | | | | | Helicopter _1_  RFDS _2_  Road _3_  Ambulance | |
| **2.1d** | What is the distance to the nearest hospital equipped for an emergency Lower Uterine Segment Caesarean Section (LUSCS)? | | | | | KM | |
| **2.1e** | What other facilities are available in town?  Primary School  Secondary School  Shop  Hospital with inpatient beds  Registered Child Care Service  Government Services (e.g., Centrelink Office) | | | | | Yes _1_ No _2_  Yes _1_ No _2_  Yes _1_ No _2_  Yes _1_ No _2_  Yes _1_ No _2_  Yes _1_ No _2_ | |
| **2.2a** | Enter total number of persons in the service area | | | | |  | |
| **2.2b** | Enter percent of females /Aboriginal and Torres Strait Islander females in the service area | | | | | % | |
| **2.2c** | Enter percent of males/Aboriginal and Torres Strait Islander males in the service area in the service area | | | | | % | |
| **2.2d** | Enter percent of Aboriginal and Torres Strait Islander people in the service area | | | | | % | |
| **2.2e** | Median age of population in the service area | | | | | *Mdn* | |
| **2.2f** | % aged 0-14 yrs % aged 15-64 yrs % aged 65+ yrs | | | | |  | |
| **2.3** | Main Industry/employment *(specify)* | |  | | | % | |
| **2.3a** | Please list the main industries/employment sources in town: | | | | | | |
| **2.3b** | Is main income from Centrelink or royalties? | | | | | | |
| **2.4** | Index of Relative Socio-Economic Disadvantage (IRSD) decile, if known?: | | | |  | | |
| **2.5** | Percent of persons employed full time | | | | | % | |
| **2.6** | Median weekly personal income | | | | | *Mdn* | |
|  | **Section Three: Estimate of Current Service Population** | | | | |  | |
| **3.1** | **Estimated number of Aboriginal &/or Torres Strait Islander people in the service area** | | | | |  | |
| **3.2** | Health Service clients. Please enter the number of: | | | | |  | |
|  | Aboriginal &/or Torres Strait Islander clients | | | | |  | |
|  | Non-Aboriginal &/or Torres Strait Islander clients | | | | |  | |
| **3.3** | **Please enter the number of clients who the Health Service considers to be Active Clients (e.g., has visited the service at least once in the previous 12 months).** | | | | |  | |
|  | Aboriginal &/or Torres Strait Islander clients | | | | |  | |
|  | Non-Aboriginal &/or Torres Strait Islander clients | | | | |  | |
| **3.4** | **How does your Health Service determine who is an active client?**  *Circle one option. If 1, enter numbers in the blank spaces below* | | | | |  | |
|  | At least ____ visits to the health centre with the past ____ years | | | | | 1 | |
|  | Geographical area or primary place of residence | | | | | 2 | |
|  | Other *(please specify)* | | | | | 3 | |
|  | No clear definition | | | | | 4 | |
| **3.5** | **How many active clients have Type 2 Diabetes Mellitus?**  **What is the average number of births per year?** | | | | |  | |
| **3.6** | **Are there seasonal variations in the Health Centre population?** | | | | | Yes _1_ No _2_ | |
| **3.6a** | If yes, please specify when and how the population varies? | | | | | | |
| **3.7** | **What other health facilities are available in the region?**  Number of hospitals Number of hospital beds  Number of clinics Type of clinic: | | | | | Private  Government  ACCHS | |
|  | **Section 4: Clinic-based staff (does not include visiting staff)** | | | | | | |
| **4.1** | **How many regular clinic-based staff positions have existed over the past 12 months:**  *Please enter the number of positions in which staff have actually been employed (full time or part time) ineach of the roles listed below over the past 12 months. This refers to the number of positions actually*  *filled, not the number of different staff members that may have been employed in the same position over the course of the year.* | | | | | | |

|  | **Aboriginal &/or Torres Strait Islander** | | **Non-Aboriginal &/or**  **Torres Strait Islander** | |
| --- | --- | --- | --- | --- |
|  | **Full time*** | **Part time^#^** | **Full time*** | **Part time^#^** |
| Managers |  |  |  |  |
| Aboriginal &/or Torres Strait Islander Health Workers OR Registered Aboriginal Health Practitioners |  |  | **-** | **-** |
| Enrolled Nurses |  |  |  |  |
| Registered Nurses |  |  |  |  |
| Clinical Nurses |  |  |  |  |
| General Practitioners (for remote communities include only resident GPs in this table) |  |  |  |  |
| Other clinical staff (e.g. allied health workers, counsellors) |  |  |  |  |
| Administrative Staff |  |  |  |  |
| Other non-clinical staff (e.g. drivers, cleaners) |  |  |  |  |

* Full time - in this column include numbers of staff who have been in full time positions over the full 12 month period

**#** Part time - in this column include numbers of staff who have been in part time positions, or positions that have only been

filled for part of the past 12 months

| **4.1a** | When compared with the last 12 months is the current workforce more, less or about the same? |  |
| --- | --- | --- |
| **4.2** | **The Manager of the PHC Centre is located:** |  |
|  | Onsite | Yes _1_ No _2_ |
|  | Off site | Yes _1_ No _2_ |

|  | **Section 5: Regular visiting staff** |
| --- | --- |
| **5.1** | **Which regular (at least once a month) visiting staff provide a regular visiting service to the health service (and have operated over the past 12 months):**  *Please enter the number of staff in each of the roles listed below.* |

|  | **Aboriginal &/or Torres Strait Islander** | **Non-Aboriginal &/or**  **Torres Strait Islander** |
| --- | --- | --- |
| Aboriginal &/or Torres Strait Islander Health Workers OR Registered Aboriginal Health Practitioners |  | - |
| Enrolled Nurses |  |  |
| Registered Nurses |  |  |
| Clinical Nurses |  |  |
| Specialists (e.g. physician, paediatrician, etc) |  |  |
| Other clinical staff (e.g. allied health workers, diabetes educators, counsellors) |  |  |
| Administrative Staff |  |  |
| Other(s) (please specify) _______________________  ___________________________________________ |  |  |

| **5.2** | **Are regular visiting staff employed by:** |  |
| --- | --- | --- |
|  | Health Board | Yes _1_ No _2_ |
|  | PHC Service | Yes _1_ No _2_ |
|  | Government | Yes _1_ No _2_ |

| **5.2a** | **Which services are routinely provided and by whom?** *(please tick the relevant boxes)* | | |
| --- | --- | --- | --- |
|  | |  |  |
|  | | Clinic Staff | Visiting Teams |
| Primary health care | |  |  |
| Antenatal and postnatal care | |  |  |
| Child health checks | |  |  |
| Women’s health | |  |  |
| Emergency and after hours care | |  |  |
| Sexual health | |  |  |
| Mental health/social and emotional wellbeing | |  |  |
| Drug and alcohol services | |  |  |
| Chronic disease care | |  |  |
| Oral health services | |  |  |
| Allied health services | |  |  |

|  | **Section 6: Retention, vacancy and turnover rates** | |
| --- | --- | --- |
| **6.1** | **Number of total current staff:**  *The total number of persons on the payroll who worked or received pay as of the last twelve months. All full-time, part-time, permanent, short-term, seasonal, salaried, and hourly employees should be included (Include GPs who work at the Service but who are self-employed sole contractors).* |  |
| **6.1a** | **Number of full-time equivalent staff:** |  |
| **6.2** | **Number of total vacant positions:**  *A vacant position means that 1) a specific full-time or part-time position exists on the organisational chart and there is work available for that position; 2) work could start within 30 days and; 3) the employer is actively recruiting from outside the Health Centre to fill the position.* |  |
| **6.3** | **Total number of employees (full or part-time) who have worked in the Health Service for 12 months or longer:** |  |
| **6.4** | **Total number of employees who have worked in the Health Service in the last 12 months:** |  |
| **6.5** | **Total number of terminations of employment in 2017?**  **Employee resignation:________**  **Employee retirement________**  **Employer dismissal_______**  **Redundancy______**  **End of fixed term/casual contract______** |  |
| **6.6** | **Retention rate:**  *Divide the total number of employees who have worked in the Health Centre for 12 months or longer as of 31 December 2017 by the total number of employees during calendar year 2017.* | % |
| **6.7** | **Vacancy rate:**  *Divide the total number of vacancies as of 31 December 2017 by the total number of established positions as of 31 December 2017.* | % |
| **6.8** | **Turnover rate:**  *Divide the total number of terminations in 2017 by the total number of employees as of 31 December 2017.* | % |

**Qualitative Interview schedule for PHC centre managers and healthcare providers**

Date: …….../………../………..

Interviewer’s name: ……………………………………………………………….

Name of facility.................................................................................................

Name of person being interviewed……………………………………………………

Has interviewee been given an information sheet and signed a consent form? Yes No

_If no, do not proceed_

Thank you for your time and participation in this project. This service has chosen to take part in this project due to its commitment to improving quality of care. We are interested in your views about how quality improvement works at the service, what works well and what might limit improvement. For the purposes of this study, quality improvement means broad service improvement to achieve a desired outcome/improve quality of care.

Do you have any questions before we start?

1. **Background**

What is your current role in service?

How long have you worked at the service

How long have you been working in remote areas?

1. **Tell me about how quality improvement works at [name of service]**

Prompts:

What do you think quality improvement means?

Who leads the implementation of quality improvement initiatives - Managers/execs/ board?

What quality improvement initiatives have you seen and who led them?

Can you describe the culture of quality improvement?

Is it linked with accreditation? [explain how this links to quality improvement]

Can you tell about how quality improvement is resourced?

1. **How are quality improvement audits done at [name of service]?**

Prompts:

- What is your role, if any, in the auditing process?
- Have you been involved in actions resulting from audits?
- Who manages and oversees quality improvement here?
- Are you involved in goal setting and objectives?

1. **Tell me about the workforce at [name of health service]? How is it now? What about in the past?**

Prompts:

- What strategies do you use to promote a stable and skilled workforce?
- What training have you received in quality improvement?
- How do you train and skill up your staff? [At local level? What about regional level?]
- How do you train and support staff who are unfamiliar with Aboriginal/Torres Strait Islander community cultures?
- What about locum/backfill support when staff go on leave or resign?
- Are there things about staff support that are done well?
- Is there anything that could be done better?

1. **How is a quality improvement focus encouraged and supported amongst the clinical workforce here?**

Prompts:

- - Is the whole clinical workforce involved?
- How are staff supported during CQI audits? – Training/backfill? Responding/ planning response?
  - How are goals for improvement chosen and achieved?
  - What strategies are used to ensure that the whole clinical workforce is involved?
  - Do any local or regional meetings focus on this topic?
  - How do staffing levels, confidence and experience impact on the ability to do CQI audits here?

1. **Do you feel you can influence quality improvement in this service?**

- If you have suggestions, to what degree do you think they can/will be implemented?

1. **Can you describe how information management and IT systems are used to support quality improvement?**

Prompts:

- - What works well?
  - Is there anything that could be improved?
  - Are all staff equally comfortable with dealing with data?

1. **How does the service use linkages with other services or organisations to support patient care?**

Prompts:

- 1. To what extent is communication between partners effective and facilitated (at regional and broader levels)
  2. To what extent do decisions made at regional level involve local service providers?
  3. How are partnerships valued within this service?
  4. How do you engage with other agencies and share data and knowledge about quality improvement?

1. **To what extent does the service involve the community in quality improvement?**
   1. in service planning?
   2. in service delivery?
   3. in service evaluation?
2. **How do you respond to identified quality issues in relation to improving patient care?**
3. **What strategies do you use to ensure continuity of quality improvement initiatives?**
4. **What do you think are the main barriers limiting response to quality improvement at this site? What about enablers?**
5. **Anything you would like to see or implemented to improve quality of care?**
6. **Anything else you would like to add about quality improvement in this context?**

**Thank you again for your time and participation, do you have any questions?**

**Would you like a copy of the transcript of the interview? Yes No**

**Focus Group/One to one Interview schedule for health service users**

Date: …….../………../………..

Interviewer’s name: ……………………………………………………………….

Name of facility.................................................................................................

Have all clients been given an information sheet and signed a consent form? Yes No

_If no, do not proceed_

Thank you all for your time and participation in this project. [insert name of health service]has chosen to take part in this project due to its commitment for improving the quality of care provided to the community. We are interested in your views about the care provided by [insert name of service] and what works well and not so well. Does anyone have any questions before we start?

1. Tell us about the last time you went to see a health professional/doctor/nurse/health worker at [insert name of service] ?
2. Are you happy with the care you receive from [insert name of service]?
   1. What do you like about the way things are done here?
   2. Is there anything that you don’t really like?
3. Do you feel comfortable with the staff at [insert name of service]? [prompt to elaborate on response]
   1. Do you feel safe with the staff at [insert name of service]? [prompt to elaborate on response]
   2. Do you feel the staff are respectful of your culture? [prompt to elaborate on response]
   3. Are you confident in the knowledge and skills of the staff providing your care?[prompt to elaborate on response]
   4. Do you think the notes the staff take help with providing good care?
4. What can you do to provide feedback to [insert name of service] to improve care?
   1. Who can you talk to if you have a problem with [insert name of service]?
   2. If you have a suggestion, are your ideas valued by staff at [insert name of service]?
   3. Are you involved in the planning of health services?
   4. Are you involved in deciding priorities for [insert name of service]?
5. Is [insert name of service] working well with other organisations to deliver care?
   1. Are there any partnerships that are important in providing care?
6. What changes have you seen at [insert name of service] that are positive?

Thank you all again for your time and participation, does anyone have any questions?

**Would you like a copy of the transcript of the interview? Yes No**
